# Impact of HER2 immunohistochemistry score on pathological complete response and survival in HER2-positive breast cancer treated with neoadjuvant therapy: differential outcomes by hormone receptor status

**DOI:** 10.3389/fonc.2026.1799768

**Published:** 2026-03-12

**Authors:** Hao Zhang, Xuemin Shen, Niya Kong

**Affiliations:** 1 Department of Thyroid and Breast Surgery, Wuhan Third Hospital, Tongren Hospital of Wuhan University, Wuhan, Hubei, China; 2 Department of Oncology, Jingmen Central Hospital Affiliated to Jingchu University of Technology, Jingmen, Hubei, China; 3 Department of General Surgery, Jingmen Central Hospital Affiliated to Jingchu University of Technology, Jingmen, Hubei, China

**Keywords:** breast cancer, HER2, neoadjuvant therapy, pathological complete response, prognosis

## Abstract

**Objective:**

To investigate the impact of differences in HER2 protein expression level based on immunohistochemistry (IHC) score (HER2 3+ *vs*. HER2 2+/FISH+) on the pathological complete response (pCR) rate and patient prognosis in HER2-positive breast cancer treated with neoadjuvant therapy(NAT).

**Methods:**

This retrospective study analyzed the clinicopathological data of 308 breast cancer patients with pathologically confirmed HER2-positive status (HER2 3+ or HER2 2+/FISH+) who received NAT combined with targeted therapy at our hospital from January 2017 to December 2020. The primary observation indicators were pCR rate and disease-free survival (DFS).

**Results:**

The pCR rate in the HER2 3+ group (252 patients, 81.8%) was significantly higher than that in the HER2 2+/FISH+ group (56 patients, 18.2%) (52.4% *vs*. 28.6%, P<0.001). Among targeted therapy regimens, patients in the HER2 3+ group receiving dual-target therapy with trastuzumab plus pertuzumab had a higher pCR rate compared to trastuzumab monotherapy (63.0% *vs*. 46.2%, P = 0.008), while patients in the HER2 2+/FISH+ group did not show significant benefit from dual-target therapy (31.8% *vs*. 26.5%, P = 0.774). Multivariate analysis identified HER2 IHC 3+, dual-target therapy, and hormone receptor (HR)-negative status as independent favorable factors for achieving pCR. During a median follow-up of 49 months, there was no statistically significant difference in DFS between the two groups (HER2 2+/FISH+ group 77.6% *vs*. HER2 3+ group 84.5%, P = 0.240). However, in the HR-positive subgroup, DFS was significantly better in the HER2 3+ group compared to the HER2 2+/FISH+ group (P = 0.024).

**Conclusion:**

High HER2 protein expression (IHC 3+) is a predictive factor for achieving a higher pCR rate with NAT in HER2-positive breast cancer. For HER2 2+/FISH+ patients, the benefit from the current standard dual-targeted therapy (trastuzumab + pertuzumab) is limited. For HER2 2+/FISH+ patients, the benefit from the current standard dual-targeted therapy (trastuzumab + pertuzumab) appears limited based on our data. However, due to the small sample size of this subgroup, this finding should be considered preliminary and requires validation in larger cohorts. These findings suggest limitations in current anti-HER2 therapy based on the existing HER2 classification. Future strategies should incorporate HER2 expression level and HR status to develop more precise individualized treatment plans.

## 1 Introduction

Breast cancer (BC) is one of the most common malignant tumors seriously threatening women’s health worldwide, with high incidence and mortality rates (1). According to molecular subtyping, HER2-positive breast cancer accounts for approximately 15%-20% of all breast cancers, characterized by overexpression and/or gene amplification of the Human Epidermal Growth Factor Receptor-2 (HER2) (2). As a key driver gene, HER2 is closely associated with aggressive tumor behavior and poor prognosis (3).

With the advent and development of anti-HER2 targeted drugs, such as trastuzumab and pertuzumab, the prognosis of HER2-positive breast cancer patients has been revolutionized (4). Neoadjuvant therapy (NAT) has become one of the standard treatment modalities for locally advanced HER2-positive breast cancer (5). Its aims are not only to downstage tumors to improve surgical resection rates but, more importantly, to predict long-term survival benefits by assessing the key surrogate endpoint of pathological complete response (pCR) and to provide a basis for adjusting subsequent adjuvant treatment strategies (6, 7).

Despite the significant success of anti-HER2 therapy, clinical practice has observed substantial heterogeneity in treatment response among patients even within the HER2-positive category. Currently, according to the American Society of Clinical Oncology/College of American Pathologists (ASCO/CAP) guidelines, HER2-positive status encompasses cases with an immunohistochemistry (IHC) score of 3+ and those with an IHC score of 2+ confirmed by fluorescence *in situ* hybridization (FISH) as having gene amplification (HER2 2+/FISH+) (8). However, growing evidence suggests that the intensity of HER2 protein expression (IHC score) may profoundly influence the efficacy of targeted therapy (9). Several studies indicate that patients with HER2 IHC 3+ often achieve higher pCR rates compared to HER2 2+/FISH+ patients and may derive more significant benefit from dual-targeted therapy (e.g., trastuzumab combined with pertuzumab) (10, 11). Furthermore, hormone receptor (HR) status, another crucial molecular feature, and its interaction with HER2 expression level in affecting treatment response and prognosis require further elucidation (12).

Therefore, an in-depth investigation into the impact of different HER2 expression levels (HER2 3+ *vs*. HER2 2+/FISH+) on pCR rates and survival outcomes in HER2-positive breast cancer undergoing NAT holds significant clinical importance for optimizing current treatment strategies, identifying patient subgroups with differential benefits, and advancing towards more precise individualized therapy. This study aims to systematically compare the clinicopathological characteristics, neoadjuvant treatment response, and survival outcomes between these two patient groups through retrospective analysis, and to explore the role of HR status, thereby providing more nuanced evidence for clinical decision-making.

## 2 Materials and methods

### 2.1 Study subjects

This study employed a retrospective cohort design. The subjects were female patients diagnosed with HER2-positive primary invasive breast cancer by core needle biopsy pathology at the Department of Thyroid and Breast Surgery, Wuhan Third Hospital, Tongren Hospital of Wuhan University, between January 2017 and December 2020. All patients received neoadjuvant chemotherapy combined with targeted therapy followed by radical surgery. All patients had signed informed consent, and this study was approved by the hospital’s medical ethics committee. All patients had signed informed consent, and this study was approved by the hospital’s medical ethics committee.

#### 2.1.1 Inclusion criteria

(1) Female patients; (2) Preoperative pathology confirming primary invasive breast cancer with HER2-positive molecular subtype; (3) HER2-positive defined as: immunohistochemistry (IHC) score of 3+, or IHC score of 2+ with gene amplification confirmed by fluorescence *in situ* hybridization (FISH) (HER2/CEP17 ratio ≥2.0 or HER2 gene copy number ≥6.0/nucleus);(4) Received at least 4 cycles of neoadjuvant chemotherapy combined with anti-HER2 targeted therapy;(5) Clinical stage II-III, without evidence of distant metastasis;(6) Underwent radical breast cancer surgery at our hospital after NAT;(7) Received standard adjuvant treatment postoperatively;(8) Complete clinical and follow-up data available.

#### 2.1.2 Exclusion criteria

(1) Male breast cancer; (2) Bilateral breast cancer; (3) Inflammatory breast cancer; (4) Recurrent or metastatic breast cancer; (5) Pregnancy-associated or lactational breast cancer;(6) Concurrent other primary malignant tumors; (7) Change of neoadjuvant treatment regimen or failure to complete the planned course; (8) Severely missing clinical data.

### 2.2 Data collection and methods

#### 2.2.1 Collection of clinicopathological data

The following data were collected from the hospital electronic medical record system: Demographic characteristics: age, menopausal status. Pretreatment tumor characteristics: clinical tumor size (cT stage), clinical nodal status (cN stage), histological grade. Molecular marker status: estrogen receptor (ER), progesterone receptor (PR), HER2 status, Ki-67 index. ER/PR positivity was defined as nuclear staining in ≥1% of tumor cells. Ki-67 was categorized using a cut-off of 30%, with >30% defined as high expression. HER2 status detection and determination strictly followed contemporaneous ASCO/CAP guidelines.

Treatment regimens: Neoadjuvant chemotherapy regimens (e.g., taxane combined with anthracycline, taxane combined with carboplatin, etc.), targeted therapy regimens (trastuzumab monotherapy or dual-target therapy with trastuzumab plus pertuzumab).

post-treatment pathological assessment: Postoperative pathological nodal stage (ypN), assessment of tumor cell regression in the primary site using the Miller-Payne (MP) grading system. Pathological complete response (pCR) was defined as the absence of invasive carcinoma in the primary breast site (allowance for ductal carcinoma *in situ*) and no metastasis in axillary lymph nodes (ypT0/is, ypN0).

#### 2.2.2 Follow-up

Follow-up was conducted through outpatient review records, inpatient medical records, and telephone interviews. The follow-up cutoff date was December 2023. The primary endpoint was disease-free survival (DFS), defined as the time from the date of surgery to the first occurrence of any of the following events: local recurrence, regional lymph node recurrence, distant metastasis, contralateral breast cancer, or death from any cause. Patients without events at the last follow-up were censored on that date.

### 2.3 Statistical analysis

Data analysis was performed using SPSS version 27.0 statistical software. Continuous variables conforming to normal distribution were presented as mean ± standard deviation and compared between groups using independent samples t-test; otherwise, the median was used. Categorical data were presented as frequency (percentage) and compared between groups using χ² test or Fisher’s exact test. Analysis of factors influencing efficacy: Binary logistic regression model was used to analyze independent factors affecting pCR, calculating odds ratios (OR) and their 95% confidence intervals (CI). Survival analysis: Kaplan-Meier method was used to plot survival curves, with between-group comparisons using the Log-rank test. Cox proportional hazards regression model was used for univariate and multivariate analysis to explore independent prognostic factors affecting DFS, calculating hazard ratios (HR) and their 95% CI. All statistical analyses were two-sided, with p<0.05 considered statistically significant.

## 3 Results

### 3.1 Patient baseline characteristics

A total of 308 HER2-positive breast cancer patients meeting the inclusion criteria were enrolled and divided into two groups based on HER2 IHC expression level: HER2 2+/FISH+ group (56 patients, 18.2%) and HER2 3+ group (252 patients, 81.8%). The overall pCR rate was 48.1% (148/308). Specifically, the pCR rate was 28.6% (16/56) in the HER2 2+/FISH+ group and 52.4% (132/252) in the HER2 3+ group. The difference in pCR rates between the two groups was statistically significant (P < 0.001).

There were no statistically significant differences between the two groups regarding age, menopausal status, initial tumor size (cT stage), histological grade, or Ki-67 expression status (P > 0.05). However, regarding hormone receptor expression, the HER2 2+/FISH+ group had significantly higher rates of ER positivity (76.8% *vs*. 31.3%) and PR positivity (41.1% *vs*. 11.5%) compared to the HER2 3+ group (P < 0.001). Furthermore, the proportion of patients with negative axillary lymph nodes (ypN0) after NAT was significantly higher in the HER2 3+ group than in the HER2 2+/FISH+ group (73.4% *vs*. 58.9%, p < 0.001). Details are shown in **Table 1**.

**Table 1:** Comparison of clinicopathological characteristics between HER2 2+/FISH+ and HER2 3+ groups [n(%)].

Clinicopathological feature	HER2 2+/FISH+ (n=56)	HER2 3+ (n=252)	p-value
Age			0.084
<55 years	52 (92.9%)	204 (81.0%)	
≥55 years	4 (7.1%)	48 (19.0%)	
Menopausal Status			0.224
Premenopausal	43 (76.8%)	171 (67.9%)	
Postmenopausal	13 (23.2%)	81 (32.1%)	
Initial Tumor Size (cT)			0.158
T1 (<2 cm)	2 (3.6%)	22 (8.7%)	
T2 (2–5 cm)	51 (91.1%)	200 (79.4%)	
T3 (>5 cm)	3 (5.4%)	25 (9.9%)	
T4	0 (0%)	5 (2.0%)	
Histological Grade			0.369
Grade 2	49 (87.5%)	200 (79.4%)	
Grade 3	3 (5.4%)	50 (19.8%)	
Unknown	4 (7.1%)	2 (0.8%)	
ER Status			<0.001
Negative	13 (23.2%)	173 (68.7%)	
Positive	43 (76.8%)	79 (31.3%)	
PR Status			<0.001
Negative	33 (58.9%)	223 (88.5%)	
Positive	23 (41.1%)	29 (11.5%)	
Ki-67 Index			0.248
≤30%	29 (51.8%)	109 (43.3%)	
>30%	27 (48.2%)	143 (56.7%)	
Postoperative Nodal Status (ypN)			<0.001
ypN0	33 (58.9%)	185 (73.4%)	
ypN1	15 (26.8%)	46 (18.2%)	
ypN2	6 (10.7%)	13 (5.2%)	
ypN3	2 (3.6%)	8(3.2%)	
Pathological Complete Response (pCR)			<0.001
No	47 (83.9%)	141 (56.0%)	
Yes	9 (16.1%)	111 (44.0%)	

### 3.2 Comparison of neoadjuvant efficacy by different treatment regimens

#### 3.2.1 Different chemotherapy regimens

Among the main neoadjuvant chemotherapy regimens (taxane combined with anthracycline, taxane combined with carboplatin), the pCR rates were significantly higher in the HER2 3+ group compared to the HER2 2+/FISH+ group (Taxane+Anthracycline: 47.5% *vs*. 29.8%, P = 0.020; Taxane+Carboplatin: 60.3% *vs*. 14.3%, P = 0.026). The number of patients receiving taxane monotherapy was small, and no statistical difference in pCR rate was observed between the two groups (P = 1.000). Details are shown in **Table 2**.

**Table 2:** Comparison of pCR rates by different chemotherapy regimens in patients with different HER2 expression levels [n(%)].

Chemotherapy regimen	HER2 2+/FISH+ (n=56)	HER2 3+ (n=252)	p-value
Taxane + Anthracycline	(n=47)	(n=179)	0.020
non-pCR	33 (70.2%)	94 (52.5%)	
pCR	14 (29.8%)	85 (47.5%)	
Taxane + Carboplatin	(n=7)	(n=58)	0.026
non-pCR	6 (85.7%)	23 (39.7%)	
pCR	1 (14.3%)	35 (60.3%)	
Taxane Monotherapy	(n=2)	(n=15)	1
non-pCR	1 (50.0%)	6 (40.0%)	
pCR	1 (50.0%)	9 (60.0%)	

#### 3.2.2 Different targeted regimens

The pCR rate in the HER2 3+ group was significantly better than that in the HER2 2+/FISH+ group, regardless of whether trastuzumab monotherapy or combination therapy with pertuzumab was used (Monotherapy: 46.2% *vs*. 26.5%, P = 0.031; Dual-target: 63.0% *vs*. 31.8%, P = 0.004). Further analysis showed that only in the HER2 3+ group did dual-target therapy significantly increase the pCR rate compared to monotherapy (63.0% *vs*. 46.2%, P = 0.008), while in the HER2 2+/FISH+ group, dual-target therapy did not show a significant advantage over monotherapy (31.8% *vs*. 26.5%, P = 0.774). Details are shown in **Table 3**.

**Table 3:** Comparison of pCR rates by different targeted regimens in patients with different HER2 expression levels [n(%)].

Targeted therapy regimen	HER2 2+/FISH+ (n=56)	HER2 3+ (n=252)	p-value (between groups)
Trastuzumab Monotherapy (H)	(n=34)	(n=171)	0.031
non-pCR	25 (73.5%)	92 (53.8%)	
pCR	9 (26.5%)	79 (46.2%)	
Trastuzumab + Pertuzumab (H+P)	(n=22)	(n=81)	0.004
non-pCR	15 (68.2%)	30 (37.0%)	
pCR	7 (31.8%)	51 (63.0%)	
p-value (Within Group Comparison)	0.774	0.008	

### 3.3 Multivariate logistic regression analysis of factors influencing pCR

Variables potentially significant in univariate analysis (age, tumor size, histological grade, HER2 expression level, targeted therapy regimen, HR status, Ki-67) were included in the multivariate logistic regression model. Results showed that HER2 IHC 3+. (*vs*. 2+/FISH+, OR = 2.124, 95% CI: 1.112 - 4.058, P = 0.022), receiving dual-target therapy (*vs*. monotherapy, OR = 1.868, 95% CI: 1.131-3.086, P = 0.015), and HR-negative status (*vs*. HR-positive, OR = 0.381, 95% CI: 0.236-0.614, P < 0.001) were independent favorable factors for achieving pCR. Details are shown in **Table 4**.

**Table 4:** Multivariate logistic regression analysis of factors influencing pathological complete response (pCR) after neoadjuvant therapy.

Variable	Reference group	OR	95% CI	p-value
Age (≥55 years)	<55 years	0.726	0.440 – 1.200	0.212
Tumor Size (T1)	T1	1.347	0.488 – 3.719	0.565
Tumor Size (T2)	T1	0.891	0.522 – 1.520	0.672
Histological Grade (Grade 3)	Grade 2	0.663	0.378 – 1.162	0.151
HER2 IHC Score (3+)	2+/FISH+	2.214	1.112 - 4.058	0.022
Targeted Therapy (Dual H+P)	Monotherapy H	1.868	1.131 – 3.086	0.015
HR Status (Negative)	Positive	0.381	0.236 – 0.614	<0.001
Ki-67 (>30%)	≤30%	1.273	0.766 – 2.116	0.351

An OR < 1 indicates a protective factor (reducing the risk of pCR), while an OR > 1 indicates a risk factor (increasing the risk of pCR). HER2 IHC 3+, dual-target therapy, and HR-negative status were identified as independent predictors of pCR in this model.

### 3.4 Subgroup analysis: impact of HR status on efficacy

In the HER2 2+/FISH+ group, pCR rates were low and showed no significant difference regardless of HR status (HR-positive: 28.9% *vs*. HR-negative: 27.3%, P = 1.000). In contrast, within the HER2 3+ group, HR-negative patients had a significantly higher pCR rate than HR-positive patients (64.5% *vs*. 36.9%, P < 0.001). Further analysis revealed that among HR-negative HER2 3+ patients, dual-target therapy yielded a significantly higher pCR rate than monotherapy (84.8% *vs*. 54.7%, P = 0.014); however, among HR-positive HER2 3+ patients and all HER2 2+/FISH+ patients, dual-target therapy did not significantly improve pCR rates compared to monotherapy (P > 0.05). Details are shown in **Table 5**.

**Table 5:** Impact of HR status on pCR rates in patients with different HER2 expression levels and subgroup analysis by targeted therapy regimen. .

Group	HR Status	pCR Rate (%)	p-value (HR comparison)	Targeted regimen	Subgroup pCR rate (%)	P-value (regimen comparison)
HER2 2+/FISH+	HR-positive	28.9 (13/45)	1	Mono H	28.1 (9/32)	0.678
				Dual H+P	30.8 (4/13)	
	HR-negative	27.3 (3/11)		Mono H	16.7 (1/6)	0.431
				Dual H+P	40.0 (2/5)	
	Total	28.6 (16/56)		Mono H	34.2 (13/38)	0.774
				Dual H+P	44.4 (8/18)	
HER2 3+	HR-positive	36.9 (41/111)	<0.001	Mono H	32.9 (25/76)	0.222
				Dual H+P	45.7 (16/35)	
	HR-negative	64.5 (91/141)		Mono H	54.7 (52/95)	0.014
				Dual H+P	84.8 (39/46)	
	Total	52.4 (148/252)		Mono H	45.0 (77/171)	0.008
				Dual H+P	67.9 (55/81)	

### 3.5 Survival analysis results

#### 3.5.1 Recurrence/metastasis and DFS

**Figure 1:**
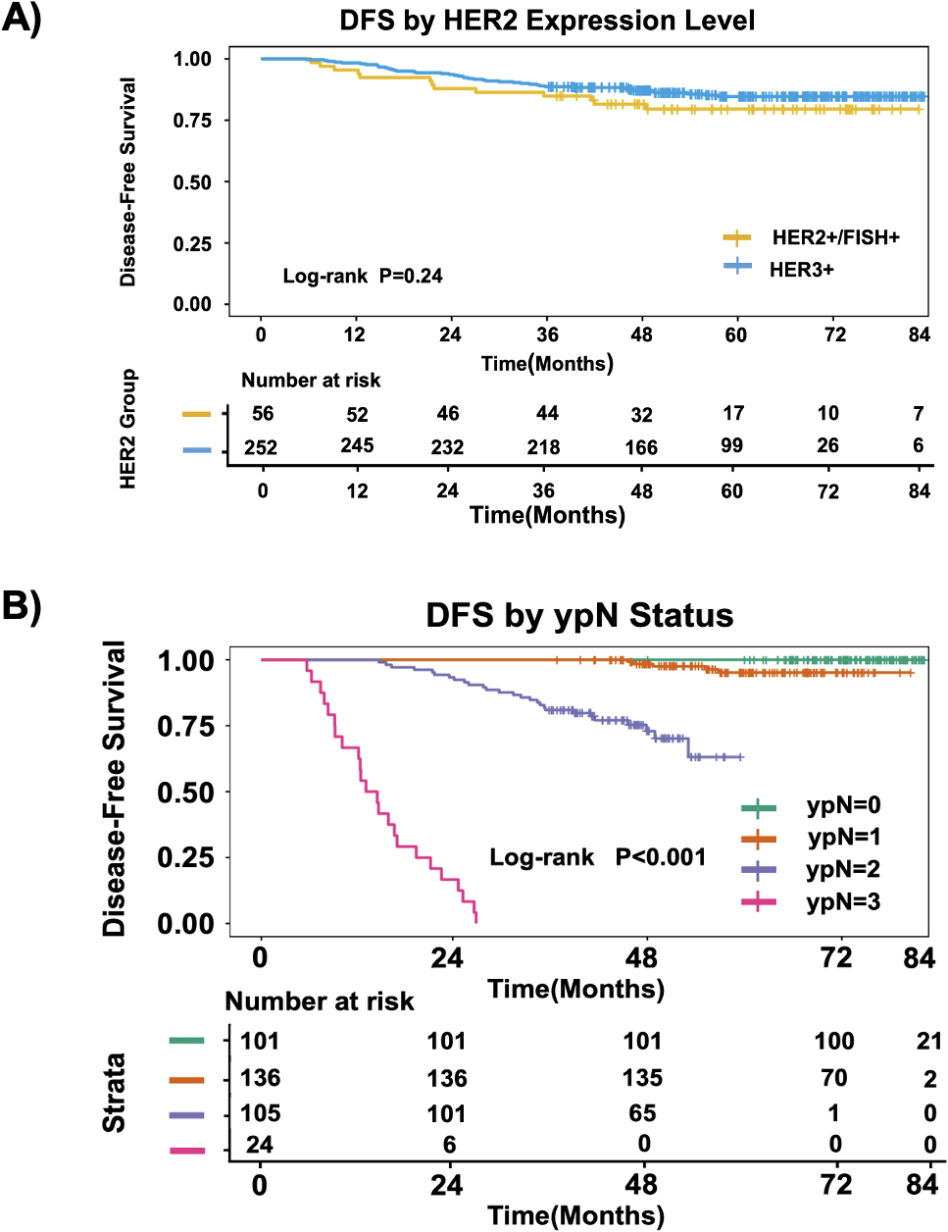
Kaplan–Meier analysis of DFS stratified by HER2 expression level **(A)** and by postoperative nodal status (ypN) **(B)**.

The median follow-up time was 49 months (range: 36–84 months). By the end of follow-up, 47 patients experienced recurrence/metastasis. The recurrence/metastasis rate was 19.6% (11/56) in the HER2 2+/FISH+ group and 14.3% (36/252) in the HER2 3+ group, with no statistically significant difference (P = 0.201). The 3-year disease-free survival (DFS) rates were 77.6% and 84.5% for the two groups, respectively. Kaplan-Meier survival curves and Log-rank test showed no statistically significant difference in DFS between the two groups (P = 0.240) (**Figure 1A**).

Further Cox regression analysis was performed on HR-positive patients (n=148). Univariate analysis showed that failure to achieve pCR with NAT, postoperative positive lymph nodes (ypN+), and HER2 IHC 2+/FISH+ status were unfavorable factors for DFS. However, in multivariate analysis, only postoperative positive nodal status (ypN1-3) was an independent risk factor for recurrence/metastasis in HR-positive HER2-positive breast cancer patients (P< 0.001) (**Figure 1B**), while the influence of HER2 expression level was no longer statistically significant(P = 0.240).

#### 3.5.2 Analysis of prognostic factors

**Figure 2:**
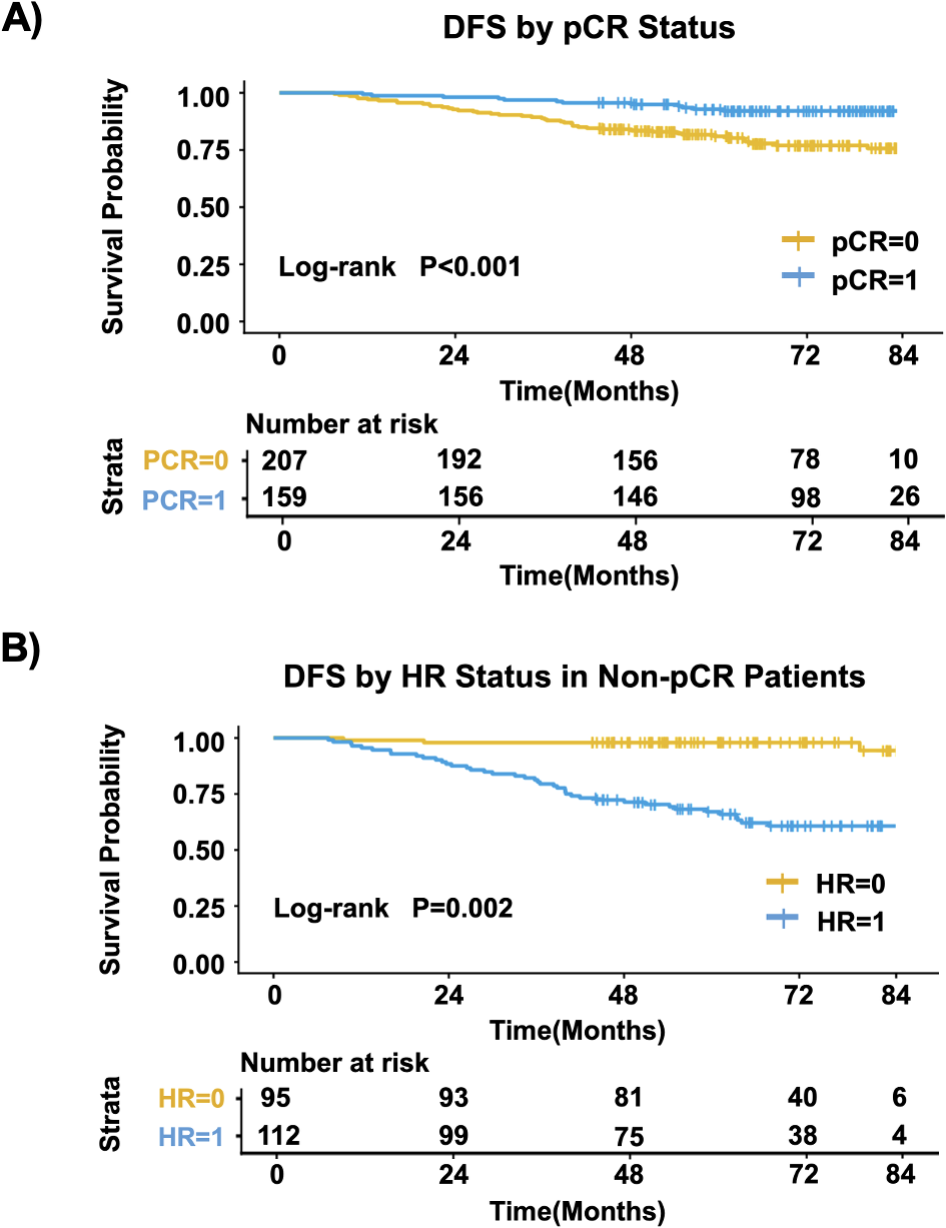
Kaplan–Meier analysis of DFS. **(A)** Comparison between patients achieving pCR and those without pCR in the overall cohort. **(B)** Comparison between HR-positive and HR-negative patients in the non-pCR subgroup.

In the overall population, patients achieving pCR had significantly better DFS than those who did not (P < 0.001) (**Figure 2A**). In the subgroup of patients who did not achieve pCR, HR-positive patients had significantly better DFS than HR-negative patients (P = 0.002) (**Figure 2B**).

#### 3.5.3 DFS comparison stratified by HR status

**Figure 3:**
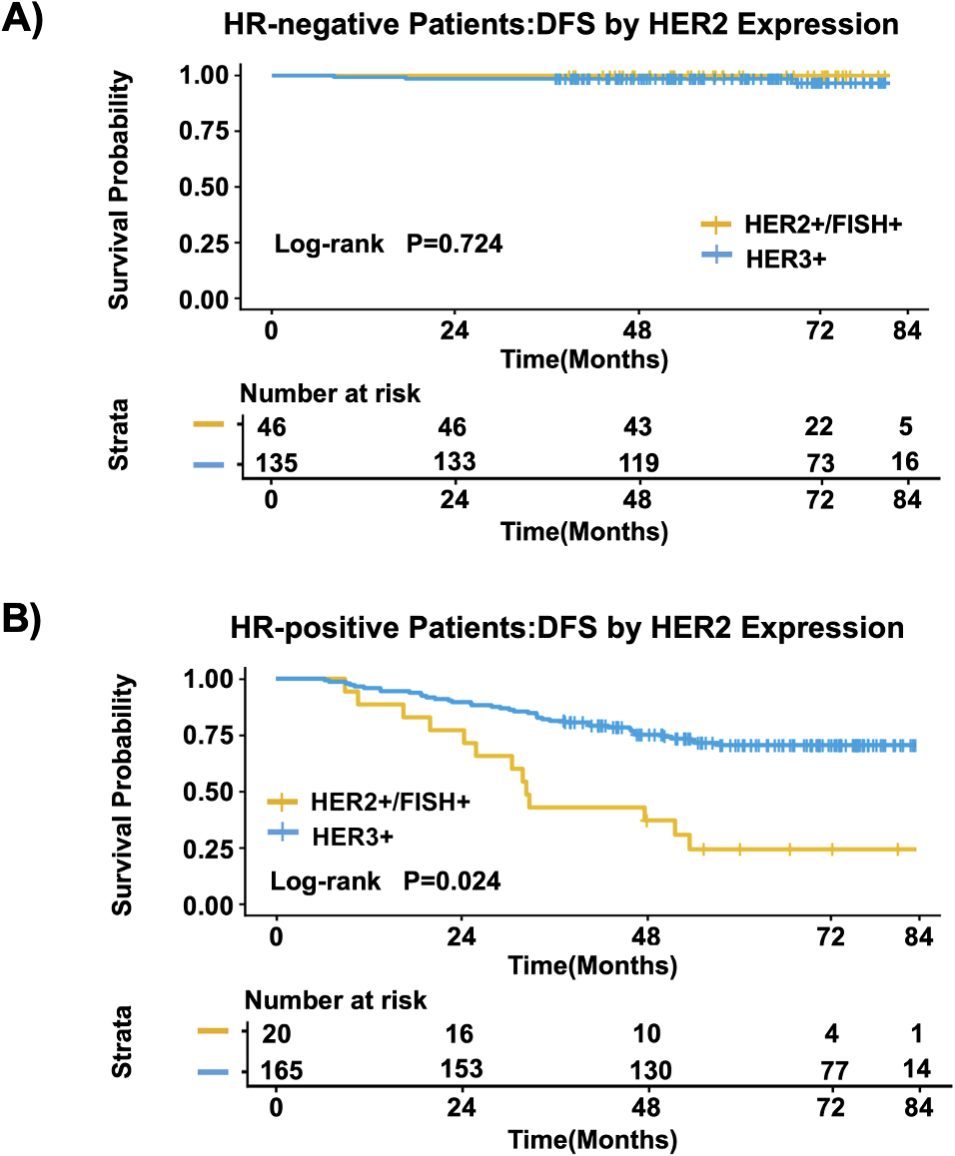
Kaplan-Meier curve analysis of DFS among HR-negative patients **(A)** and among HR-positive patients **(B)**.

Among HR-negative patients, there was no significant difference in DFS between the HER2 3+ group and the HER2 2+/FISH+ group (P = 0.724) (**Figure 3A**). However, among HR-positive patients, the HER2 3+ group had significantly better DFS than the HER2 2+/FISH+ group (Log-rank p = 0.024) (**Figure 3B**).

## 4 Discussion

This retrospective analysis of 308 HER2-positive breast cancer patients receiving NAT confirms that HER2 protein expression level is a key factor influencing treatment response. The results show that pCR rate was significantly higher in HER2 IHC 3+ patients compared to HER2 2+/FISH+ patients (52.4% *vs*. 28.6%), and that HER2 IHC 3+, dual-target therapy, and HR-negative status were independent favorable factors for achieving pCR. This finding aligns with previous studies indicating that HER2 protein overexpression (IHC 3+) is a strong predictor of sensitivity to anti-HER2 targeted therapy (13–15). The underlying mechanism may be that IHC 3+ signifies a higher density of HER2 receptors on the tumor cell surface, allowing monoclonal antibodies targeting HER2 (such as trastuzumab and pertuzumab) to bind more effectively, inhibit downstream oncogenic signaling pathways, and more fully activate antibody-dependent cell-mediated cytotoxicity (5). In contrast, HER2 2+/FISH+ tumors, despite having gene amplification, exhibit weaker or heterogeneous protein expression, potentially reducing the binding efficiency of targeted drugs and thus diminishing efficacy. This study, along with prior research, observed that HER2 2+/FISH+ tumors are often associated with a higher rate of HR positivity (16, 17), suggesting they may possess distinct molecular biological characteristics from typical HER2-high tumors, with potentially more complex driving mechanisms. In contrast, HER2 2+/FISH+ tumors, despite having gene amplification, exhibit weaker or heterogeneous protein expression. It is plausible that this lower receptor density may lead to reduced binding efficiency of targeted antibodies, thereby contributing to the diminished efficacy observed in this subgroup. However, this mechanistic hypothesis requires validation in future studies with appropriate experimental designs, such as quantitative assessment of HER2 receptor density and its correlation with drug binding and downstream signaling inhibition.

A key finding of this study is the significant difference in the benefit of dual-targeted therapy (trastuzumab plus pertuzumab) among patients with different HER2 expression levels. In HER2 3+ patients, dual-target therapy significantly increased the pCR rate (from 46.2% to 63.0%), consistent with results from key clinical trials like APHINITY, confirming the synergistic effect of dual HER2 blockade in this population (16). However, in HER2 2+/FISH+ patients, dual-target therapy did not show superior efficacy compared to monotherapy (pCR rate: 31.8% *vs*. 26.5%). This strongly suggests that the current standard dual-target therapy strategy, based on the “binary” HER2-positive classification (positive/negative), might represent “overtreatment” or “insufficient efficacy” for the specific subgroup of HER2 2+/FISH+ patients (18). Pertuzumab, by binding to a different epitope on the HER2 receptor, primarily inhibits HER2-HER3 dimerization (5). When HER2 protein expression is limited, this dual blockade may not generate sufficient additive effects. Therefore, for HER2 2+/FISH+ patients, there is an urgent need to explore more optimal treatment strategies, such as considering combination with endocrine therapy (for HR-positive patients), switching to small-molecule tyrosine kinase inhibitors (TKIs) with different mechanisms like pyrotinib (19), or next-generation antibody-drug conjugates (ADCs) such as T-DXd (20).

The modulating effect of HR status on treatment efficacy and prognosis is particularly prominent in this study. First, HR positivity was an unfavorable factor for achieving pCR, consistent with the general perception that HER2+/HR+ breast cancer is relatively less sensitive to chemotherapy (9, 21, 22). In-depth analysis revealed that the negative impact of HR primarily existed within the HER2 3+ subgroup; whereas in the HER2 2+/FISH+ subgroup, pCR rates remained low regardless of HR status. This reveals the complex interaction between HER2 expression intensity and the HR signaling pathway (12). Second, regarding survival prognosis, a noteworthy finding was that among HR-positive patients, DFS was significantly better in the HER2 3+ group compared to the HER2 2+/FISH+ group (P = 0.024). This may be attributed to the significant survival benefit from subsequent adjuvant endocrine therapy in HER2 3+/HR+ patients even if they did not achieve pCR after effective neoadjuvant targeted therapy combined with chemotherapy (19). Conversely, HER2 2+/FISH+/HR+ patients, having poor response to NAT, may have a higher burden of residual disease, and their sensitivity to endocrine therapy might also be affected by concurrent low-level HER2 signaling (12, 23). Multivariate analysis ultimately showed that in the HR-positive population, the most critical factor determining long-term prognosis was the post-NAT axillary lymph node status, rather than HER2 expression level itself. This emphasizes the paramount importance of achieving deep pathological response (including both primary site and lymph nodes) for these patients. HR-positive patients who do not achieve pCR and have positive lymph nodes after NAT should be considered high-risk and candidates for intensified adjuvant therapy (2, 24), such as replacing trastuzumab with T-DM1 (25).

This study also has certain limitations. First, as a single-center retrospective study, selection bias is inevitable. Second, the sample size of the HER2 2+/FISH+ group was relatively small, which may affect the stability of some subgroup analysis results, particularly when evaluating the efficacy of different chemotherapy regimens. Third, the follow-up duration is insufficient to assess long-term overall survival differences. Fourth, the sample size of the HER2 2+/FISH+ group (n=56) was relatively small compared to the HER2 3+ group, which limited the statistical power for subgroup analyses, particularly when evaluating the efficacy of different chemotherapy and targeted therapy regimens within this subgroup. Therefore, findings from these subgroup analyses should be considered exploratory and require validation in larger, prospective cohorts specifically enriched for HER2 2+/FISH+ patients. Fifth, treatment-related adverse events were not analyzed due to incomplete retrospective data, which would have provided important context for evaluating the risk-benefit ratio of dual-target therapy in HER2 2+/FISH+ patients. Finally, the study did not incorporate specific numerical values of HER2 gene copy number or HER2/CEP17 ratio for continuous variable analysis, preventing an in-depth exploration of the fine-grained association between the degree of gene amplification and treatment efficacy.

In summary, this study indicates that in the NAT of HER2-positive breast cancer, HER2 protein expression level (IHC 3+ *vs*. 2+/FISH+) is an important stratification factor for predicting pCR rate. HER2 2+/FISH+ patients show limited response to current standard dual-target therapy and have relatively poorer prognosis (especially when HR-positive), representing a clinical challenge and a potential direction for precision therapy research. However, given the modest sample size of the HER2 2+/FISH+ subgroup and the retrospective nature of this study, these findings should be interpreted with caution and considered hypothesis-generating rather than definitive. They highlight a clinical challenge and suggest a potential direction for future precision therapy research, but require confirmation in larger, prospective studies specifically enriched for HER2 2+/FISH+ patients. Future prospective studies should be conducted to build more refined prognostic prediction models based on the continuous spectrum of HER2 expression (including mRNA levels, protein quantification, and intratumoral heterogeneity) combined with multi-omics information such as HR status and immune microenvironment. This will enable truly individualized stratified treatment for HER2-positive breast cancer, sparing those with high expression from overtreatment and providing intensified therapy for those with low expression/heterogeneity.

## Data Availability

The original contributions presented in the study are included in the article/supplementary material. Further inquiries can be directed to the corresponding author.
